# Genome-wide CRISPRi screen and proteomic profiling identify key genes related to ferulic acid’s antifungal activity

**DOI:** 10.1128/mbio.01909-25

**Published:** 2025-08-25

**Authors:** Ofri Levi, Rina Zuchman, Nour Sleman, Roni Koren, Hazem Khamaisi, Benjamin A. Horwitz

**Affiliations:** 1 Faculty of Sciences and Technology, Tel-Hai Academic College, Upper Galilee, Israel; 2 MIGAL - Galilee Research Institute, Kiryat Shmona, Israel; 3 Faculty of Biology, Technion - Israel Institute of Technology, Haifa, Israel; University of California Davis 8789, Davis, California, USA

**Keywords:** antifungal resistance, CRISPR interference (CRISPRi), plant-derived phenolic compounds, drug synergy, eco-friendly fungicides, fungal pathogens, ferulic acid, ergosterol biosynthesis

## Abstract

Fungal pathogens of plants must overcome host-imposed stressors, including antimicrobial small molecules. Ferulic acid (FA), a plant-derived phenolic compound, induces fungal stress and cell death. To uncover genetic determinants of FA sensitivity, we performed a genome-wide CRISPR interference (CRISPRi) screen in *Saccharomyces cerevisiae*. We confirmed that FA impairs yeast growth and triggers stress granule marker sequestration, establishing a relevant selection condition. The CRISPRi screen identified 194 genes involved in the FA-induced stress response and 12 whose repression enhanced resistance. Among them, ERG9, encoding squalene synthase, was most strongly enriched, and its repression conferred FA resistance alongside upregulation of HMG1, implicating the ergosterol biosynthesis pathway. Proteomic profiling of FA-resistant *Cochliobolus heterostrophus* strains further revealed conserved upregulation of ergosterol biosynthetic enzymes. FA also synergized with fluconazole, a known ergosterol-targeting antifungal, and enhanced susceptibility in azole-resistant *Candida albicans* strains, suggesting interference with ergosterol metabolism. *In planta*, FA exhibited dose-dependent antifungal activity, significantly reducing *C. heterostrophus* lesion formation in maize. These findings establish FA as a promising antifungal agent that targets conserved lipid biosynthesis pathways and overcomes resistance mechanisms, supporting its potential as a sustainable therapeutic and agricultural fungicide.

**IMPORTANCE:**

Fungal infections are a growing threat to human health and agriculture, with rising antifungal resistance limiting treatment options. In this study, we used a genome-wide screening approach to identify ferulic acid (FA), a naturally occurring compound found in plants, as a promising antifungal agent. FA targets the same cellular pathway as many current antifungal drugs and works especially well when combined with fluconazole, a commonly used treatment. Remarkably, FA is also effective against drug-resistant *Candida albicans* strains, offering hope for new ways to treat difficult infections. In addition to its medical potential, FA protects maize from fungal pathogens, highlighting its usefulness as a sustainable and environmentally friendly crop protectant. These results suggest that FA could be developed into a versatile antifungal agent with applications in both clinical and agricultural settings, helping address the urgent need for new strategies to overcome antifungal resistance.

## INTRODUCTION

Among the vast diversity of over 99,000 identified fungal species (1, 2), only a select few hundred are known to cause diseases in humans (3). Notable among these are *Candida* spp., *Aspergillus* spp., *Cryptococcus* spp., and *Pneumocystis jirovecii* (4, 5). Moreover, certain plant pathogenic fungi, such as *Magnaporthe oryzae*, *Botrytis cinerea*, *Puccinia* spp*.*, *Fusarium* spp*.*, *Blumeria graminis*, *Mycosphaerella graminicola*, and *Colletotrichum* spp*.*, can lead to significant crop losses (6).

To address these issues, clinical antifungal drugs and agricultural fungicides have been widely used to combat fungal pathogens (7). Clinical antifungal therapy primarily relies on three drug classes: azoles, polyenes, and echinocandins (8). Azoles inhibit the lanosterol 14-α-demethylase enzyme (*ERG11*/CYP51), causing ergosterol depletion and toxic intermediate accumulation, which impairs fungal growth and cell division (9–11). Resistance arises from modifications in the ergosterol pathway, including point mutations in *ERG11* and overexpression of target enzymes (12), as well as drug expulsion from the cell through overexpression of multidrug transporters belonging to the ATP-binding cassette (ABC) superfamily (11, 13–16). The extensive use of antifungal drugs is recognized as a major cause of worldwide antifungal resistance (17–21). Therefore, further comprehensive research efforts addressing fungal genetics and gene expression regulation mechanisms that facilitate antifungal resistance will contribute to improved treatments and patient prognosis.

Additionally, agricultural fungicides have been widely used to combat fungal pathogens and improve crop yield and quality (7). Similar to antifungal drugs, resistance to agricultural fungicides has quickly emerged. Benzimidazoles like methyl benzimidazole carbamate saw resistance due to β-tubulin mutations (22–24), while succinate dehydrogenase inhibitors and anilinopyrimidines encountered resistance via mitochondrial mutations and efflux mechanisms, respectively (25–28). Likewise, mutations in the cytochrome b gene drive resistance to Qo inhibitors (QoI) (29, 30), and morpholines face moderate risks of resistance (31, 32).

Furthermore, the use of antifungal agents in agriculture raises potential threats to human health, with reported adverse effects on endocrine, immunological, neurological, and carcinogenic systems (33). Importantly, dual-use antifungals, including azoles and the recently characterized orotomides, widely employed in agriculture, may contribute to the emergence of resistant fungal strains, posing risks to both plant and human health (34–36). Thus, developing environmentally friendly antifungal agents is a critical goal. One recent strategy is to apply small-molecule agents that enhance the activity of conventional fungicides or restore activity against resistant strains (37). Plant compounds are a likely source of such agents, and plant extracts are studied for their fungicidal potential (38, 39). Many studies employed direct-contact assays and tested these compounds on various plants like corn, wheat, soybean, chickpea, pistachio, peanut, and rice (40–45). Notably, these antifungal extracts, often containing phenolic acids, terpenes, or terpenoids, exhibit remarkable diversity (46, 47). Phenolic acids have been found to impede the growth of both filamentous fungi and yeast. While numerous studies have explored this phenomenon, a definitive mode of action remains elusive. Primarily investigated with the opportunistic human pathogen *Candida albicans* (*C. albicans*), research suggests that phenolic acids may compromise cell membrane integrity (48). Additionally, certain phenolic compounds have demonstrated the ability to trigger apoptotic pathways in *Candida* spp., thereby exerting their antifungal effects (48, 49).

In filamentous fungi, the impact of phenolic acids on fungal development and mycotoxin production has been examined in other genera. In *Aspergillus* spp., for instance, a concentration of 1 mM ferulic acid (FA) inhibits growth by 30% and reduces aflatoxin production by 50% (50). The effects of other phenolic compounds vary; for example, salicylic acid (at concentrations of 1 and 5 mM) shows no discernible impact on Aspergillus growth, whereas similar concentrations of thymol and cinnamic acid led to a 50% to 70% inhibition of growth (51).

FA, a common phenolic acid compound in plants, exhibits significant antibacterial effects (52, 53). Furthermore, FA demonstrates antifungal properties. FA exhibits potent antifungal activity against the radial growth of *Fusarium graminearum*, showing greater inhibition than other prevalent cereal phenolic acids (54, 55). Moreover, FA is a major component of maize seed pericarp extract, which suppresses *Fusarium verticillioides* growth and fumonisin B1 accumulation (56). FA’s potential applications in food safety, particularly in controlling Fusarium infections in fruits and cereal grains, have attracted considerable attention (57, 58). Moreover, Canturk et al. demonstrated a notable synergy between FA and caspofungin against *C. albicans*, suggesting that this combined treatment could offer a promising new approach to managing Candida infections (59). The inhibitory mechanism of FA, however, remains unknown.

Herein, to systematically identify genetic determinants of FA susceptibility, we employed a genome-wide CRISPRi screen in *Saccharomyces cerevisiae*. Although *S. cerevisiae* is non-pathogenic, its exceptionally high transformation efficiency, robust genetic tractability, and wealth of functional genomic tools make it an ideal model organism for large-scale genetic screens. Our data indicate that FA exposure perturbs the ergosterol biosynthesis pathway, as evidenced by the enhanced FA resistance observed upon *ERG9* repression. *ERG9* repression correlated with *HMG1/2* upregulation, suggesting a compensatory response in sterol biosynthesis. To validate and extend these findings to pathogenic fungi, we performed proteomic profiling of FA-resistant *Cochliobolus heterostrophus* strains. Comparative proteomic analysis revealed a conserved core resistance program involving upregulation of ergosterol biosynthesis enzymes. Additionally, we demonstrate a potent synergistic interaction between FA and fluconazole (FLC), significantly reducing the MIC required to inhibit *C. albicans* and *Candida parapsilosis* (*C. parapsilosis*). Notably, azole-resistant *C. albicans* strains display heightened sensitivity to FA treatment. Finally, FA showed, in a maize model *in planta*, a dose-dependent reduction in *Cochliobolus heterostrophus* lesions, effectively decreasing their count and size. These results uncover novel molecular pathways and components affected by FA and highlight its potential utility as an antifungal agent and synergistic enhancer of existing antifungal therapies.

## RESULTS

### A comprehensive genome-wide CRISPRi analysis of ferulic acid treatment

To comprehensively identify and characterize molecular pathways and resistance mechanisms associated with FA treatment, we utilized a previously established genome-wide CRISPRi library (Addgene #161829) (60). This system takes advantage of a single-plasmid inducible system expressing a single gRNA and the catalytically inactive dCas9 fused to the MXI1 transcriptional repressor (60, 61). Upon induction with anhydrotetracycline (ATc) of single gRNA expression, this CRISPRi system specifically represses the expression of the gRNA target gene.

Overall, this library consists of a vast collection of gRNAs (>51,000) targeting all *S. cerevisiae* genes, with 6 to 12 gRNAs per gene, allowing high-throughput screening. gRNA depletion indicates genes required for survival under FA stress, while gRNA enrichment reveals genes whose repression enhances FA resistance.

We first validated the response of *S. cerevisiae* to FA by assessing yeast growth under increasing FA concentrations. Growth curve analysis revealed that FA progressively inhibited *S. cerevisiae* growth in a dose-dependent manner. Specifically, FA reduced growth by 6.9%, 24.5%, 48.9%, and 88.9% at concentrations of 31.25, 62.5, 125, and 250 µg/mL, respectively. At the highest concentration tested (500 µg/mL), no *S. cerevisiae* growth was observed over 48 h, indicating strong inhibition or toxicity (Fig. S1). Fluorescence confocal microscopy of Pab1-GFP and Gus1-GFP expressing strains, both stress granule markers, revealed that FA treatment at 2.5 mM induces a dispersed and punctate localization pattern for these proteins, in contrast to their uniform distribution in the dimethyl sulfoxide (DMSO) control. This suggests that FA disrupts normal protein localization and likely induces the formation of stress granules, reflecting the cellular response to FA-induced stress. (Fig. S1). The results showed a significant reduction in *S. cerevisiae* growth rate as FA concentration increased, indicating similar sensitivity to FA as observed in *Candida* spp. and filamentous fungi (62).

To systematically identify genes influencing FA susceptibility, we conducted a genome-wide CRISPRi screen in *S. cerevisiae*, using a pooled transformant library to ensure uniform gRNA representation at the start of the experiment. We subjected approximately six million yeast cells harboring the CRISPRi library to a competitive growth assay in SC-His medium supplemented with 250 ng/mL ATc and 2.5 mM FA, alongside a DMSO-treated control. Cultures were grown to the stationary phase (O.D. ~8) and subjected to three sequential rounds of dilution and regrowth to enrich for FA-resistant populations.

**Fig 1:**
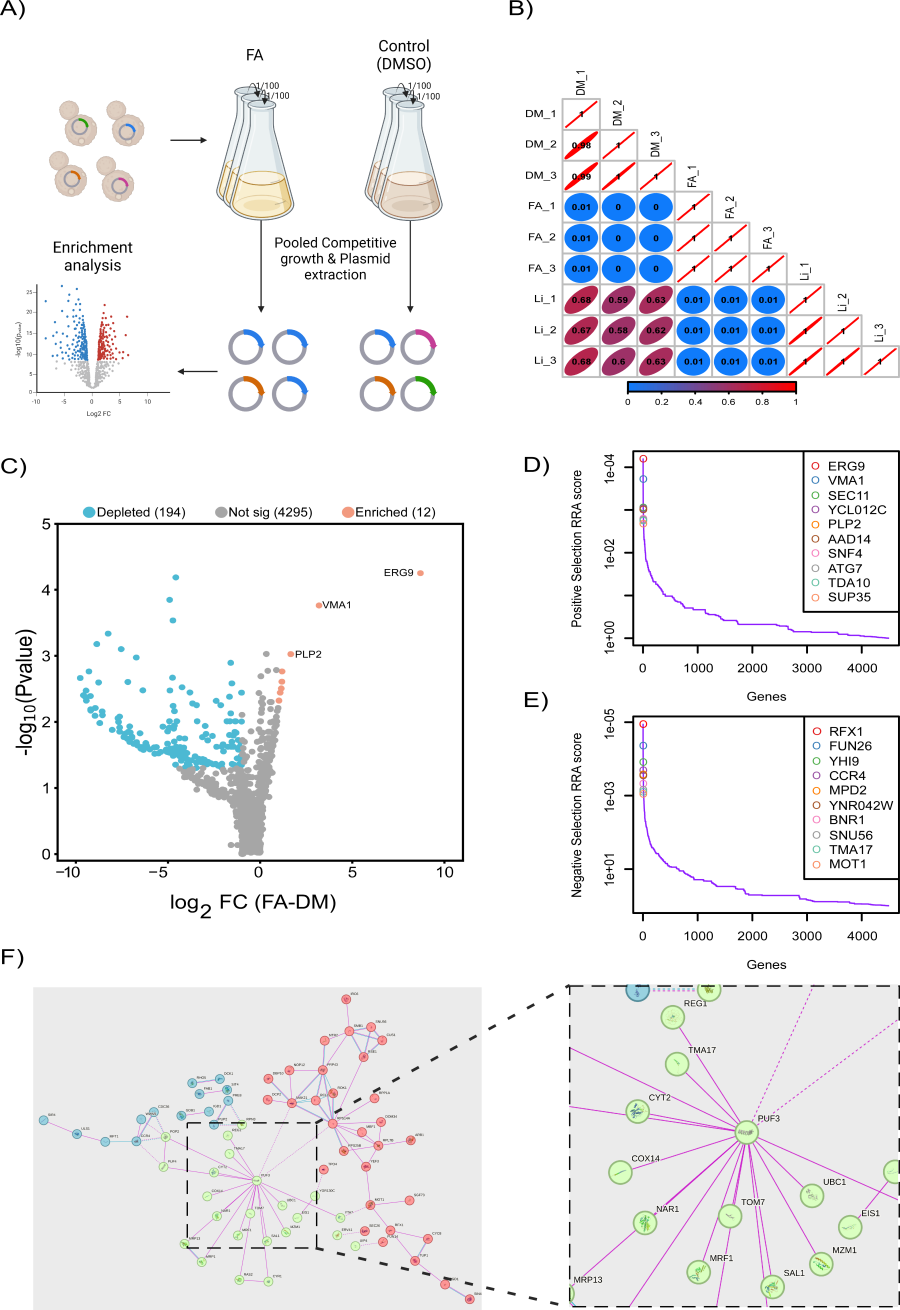
Comprehensive genome-wide CRISPRi analysis of *S. cerevisiae* response to ferulic acid. (**A**) Schematic representation of the CRISPRi library screening and competitive growth. *S. cerevisiae* cells expressing a genome-wide CRISPRi library were subjected to competitive growth in the presence of FA or DMSO (control). The dCas9-Mxi1 system, guided by gRNAs, repressed target gene transcription upon induction with ATc. Pooled cultures underwent serial dilutions (1:100), followed by plasmid extraction and sequencing to assess gRNA abundance. Enrichment analysis identified genes whose repression either increased sensitivity (depleted gRNAs) or conferred resistance (enriched gRNAs) to FA, as visualized in the volcano plot. (**B**) Correlation matrix showing Pearson correlation coefficients between biological replicates of FA-treated (FA), DMSO control (DM), and initial library samples (Li). The color scale (blue to red) indicates the strength of correlation. (**C**) Volcano plot illustrates differential gRNA abundance between FA-treated and DMSO control samples. Genes significantly depleted (blue) or enriched (orange) are highlighted, with thresholds of log2 fold change >2 or <−2 and *P* value <0.05. (**D**) Top-ranked genes sensitive to FA treatment. The top 10 genes with the highest RRA scores in negative selection. (**E**) Top-ranked genes contributing to FA resistance. The top 10 genes with the highest RRA scores in positive selection. (**F**) STRING network analysis of 194 genes depleted upon FA treatment. The network is divided into three distinct clusters: blue, green, and red. The blue cluster primarily contains genes involved in ribosomal biogenesis and RNA metabolism. The green cluster includes genes related to mitochondrial function, protein degradation, and stress responses. The red cluster comprises genes associated with mRNA processing, splicing, and transport.

To address potential biases introduced by competition among strains with differing growth rates, we included a DMSO control condition in parallel with FA treatment. This allowed us to distinguish FA-specific responses from general fitness defects. Plasmids were extracted from three independent biological replicates per condition (2.5 mM FA, DMSO control, and the initial library [Li], representing the baseline gRNA distribution in the unexposed pooled population), and amplicon sequencing was performed to quantify gRNA abundance across conditions (Fig. 1A). Following sequencing, gRNA read counts were analyzed using the MAGeCK pipeline. The abundance of each gRNA in FA-treated versus DMSO-treated populations was compared to calculate fold change and statistical significance. Gene-level scores were derived using MAGeCK’s robust rank aggregation (RRA) algorithm, which integrates data from all gRNAs targeting a given gene. Depletion of gRNAs suggests that repression of the gene reduces cellular fitness during FA exposure, whereas enrichment indicates that gene repression confers a relative fitness advantage under FA treatment. This approach enabled the identification of genes enriched or depleted under FA treatment, ensuring that observed genetic interactions were specific to FA exposure rather than artifacts arising from strain competition or general fitness defects.

Overall, 22,881 gRNAs were mapped in all three CRISPRi initial library biological repeats, with an average coverage of 4.89 gRNAs per gene (Fig. S1; Table S1). Principal component analysis showed a clear separation between FA-treated samples, DMSO controls, and the initial library, suggesting that FA treatment alters gRNA distribution patterns (Fig. S2B). The distribution of gRNA counts across different samples demonstrated that FA treatment increased variability, with FA-treated samples exhibiting a wider distribution and reduced median gRNA counts compared to the DMSO controls (Fig. S2C). A high correlation is apparent between all three FA-treated samples. Thus, the CRISPRi screen is reproducible, attested by the high correlation between biological repeats, and demonstrates specificity, as indicated by the low correlation with the DMSO control and initial library samples (Fig. 1B). Overall, 344 gRNAs targeting 194 genes appeared significantly depleted, and 12 appeared enriched upon FA treatment (fold change >2 or <−2, *P* value < 0.05) (Fig. 1C; Table S2).

The top ten depleted genes from the CRISPRi screen include RFX1 and MOT1 (transcription regulators), FUN26 (transmembrane transporter), YHI9 and MPD2 (unfolded protein response), CCR4 and SNU56 (mRNA processing), BNR1 (actin filament assembly), TMA17 (ribosomal function), and YNR042W (dubious ORF). Their reduced activity suggests these genes are crucial for managing FA-induced stress (Fig. 1F).

Moreover, functional Gene Ontology (GO) term enrichment analysis (using Gorilla [63]) among the 194 genes depleted upon FA treatment revealed enrichment of several RNA expression regulation GOs such as nuclear-transcribed mRNA catabolic process, deadenylation-dependent decay (GO:0000288), and positive regulation of RNA metabolic process (GO:0051254) (Fig. S3A; Table S3). This suggests that these pathways are important to the cellular response to FA. STRING network analysis (64) of the 194 FA-depleted genes revealed three clusters: ribosomal biogenesis and RNA metabolism (blue: CDC36, RHO5, STT4), mitochondrial function and stress responses (green: COX3, TCM1, RAS2), and mRNA processing and transport (red: NOP1, PRP43, DBP10), suggesting FA impacts protein synthesis, metabolic processes, and RNA stability. PUF3 was central in the network, with extensive interactions, highlighting its key role in the FA response. Sub-central nodes included RPL8A (RNA processing), COX3 (mitochondrial function), and CDC36 (ribosomal biogenesis). To reveal the sub-cellular localization of Puf3 upon FA treatment, a GFP-tagged PUF3 strain was used. Puf3-GFP localization showed concentrated foci under FA treatment, suggesting FA affects Puf3 dynamics and may influence its regulatory role in mitochondrial mRNA stability and stress adaptation (Fig. 1F; Fig. S3B).

FA treatment enriched several key genes, including VMA1 (vacuolar ATPase activity), SEC11 (signal peptidase complex), YCL012C (uncharacterized), PLP2 (phospholipid metabolism), AAD12 (aryl-alcohol dehydrogenase), SNF4 (Snf1 kinase regulation), ATG7 (autophagy), TDA10 (unknown function), SUP35 (translation termination), YAT2 (carnitine acetyltransferase), and RPS14A (ribosomal 40S subunit). Their increased activity suggests important roles in the *S. cerevisiae* response to FA (Fig. 1D).

### Examining the impact of ferulic acid on the ergosterol biosynthesis pathway

**Fig 2:**
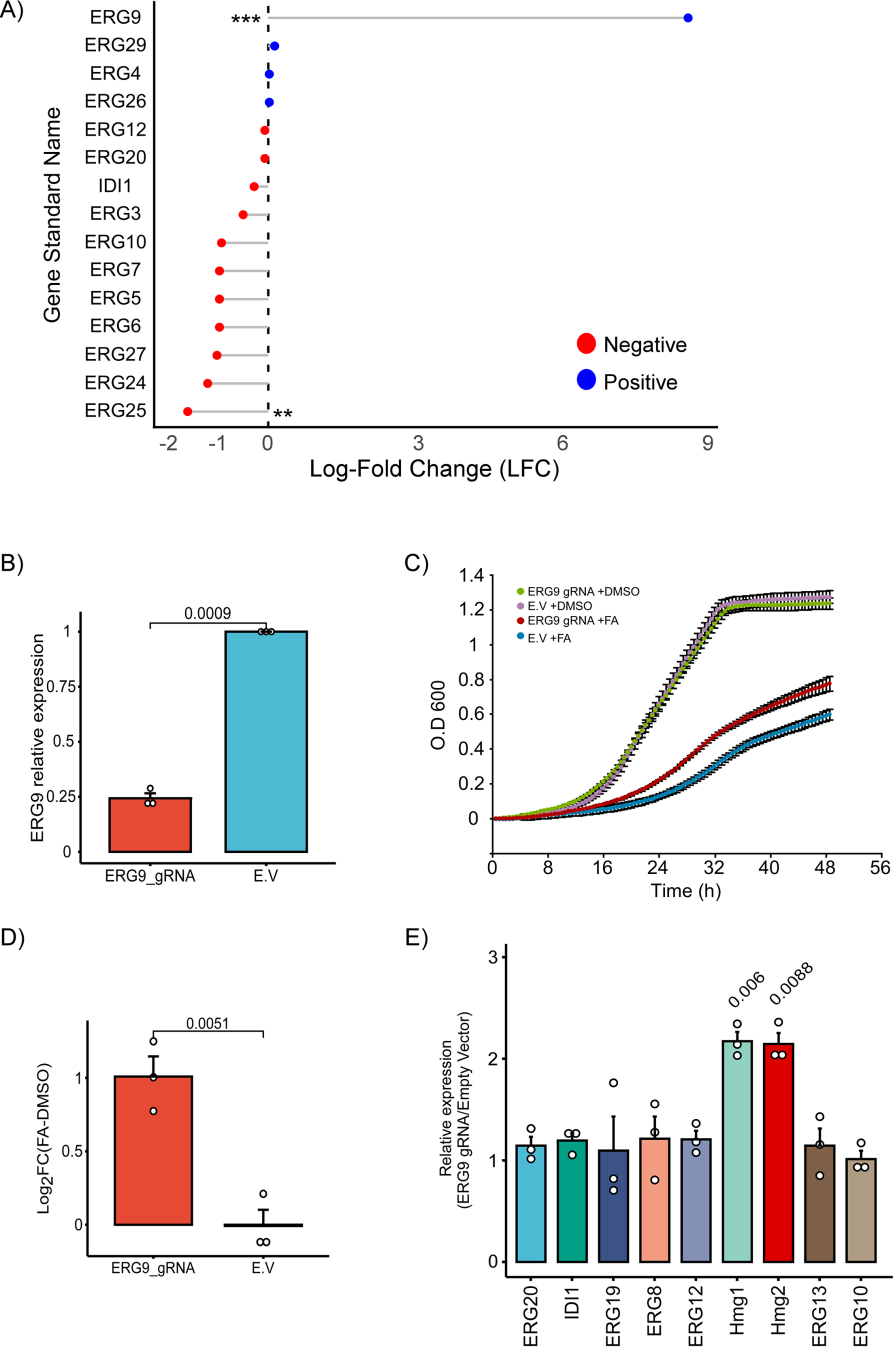
Effect of *ERG9* silencing on FA resistance and upstream enzyme expression. (**A**) Lollipop plot of ergosterol biosynthesis genes. Log2 fold change (FA-DMSO) obtained from MAGeCK test analysis. Each gene response is colored based on the direction of fold change, with red indicating a negative log-fold change and blue indicating a positive log-fold change. Asterisks denote statistical significance levels: * <0.05, ** <0.01, and *** <0.001. (**B**) Reverse transcription-quantitative polymerase chain reaction (RT-qPCR) confirmation of *ERG9* silencing. mRNAs were quantified by RT-qPCR analysis from three independent biological repeats, each with three technical repeats, normalized to ACT1 mRNA levels. (**C**) Yeast growth assay comparing the FA resistance of the *ERG9* CRISPRi strain to the empty vector (E.V.) control. Growth was monitored in the presence of DMSO and 1.5 mM FA over 48 h. (**D**) Quantitative analysis of Yeast drop assay. Log2 fold-change (FA-DMSO) in the *ERG9* CRISPRi strain compared to the empty vector. Error bars represent the standard error of the mean (SEM) from three independent biological repeats, and the *P* value was calculated by the dependent samples one-tailed *t*-test. (**E**) Expression levels of nine upstream enzymes in the ergosterol biosynthesis pathway following *ERG9* silencing. mRNAs were quantified by RT-qPCR analysis and normalized to ACT1 mRNA levels and empty vector expression levels. The histogram presents the quantification of three independent biological repeats, each with three technical repeats. *P* values were calculated by the dependent samples one-tailed *t*-test.

The genome-wide CRISPRi screen conducted to identify genes involved in the response to FA revealed significant insights into the connection between FA resistance and the ergosterol biosynthesis pathway (Fig. 2A). A top-ranked gRNA that increases FA resistance is *ERG9* (log2 fold change = 8.6936, *P* value = 5.61E-05) (Fig. 1C and E; Table S2). This suggests that *ERG9* silencing can increase FA resistance. *ERG9* encodes squalene synthase, an essential enzyme in sterol biosynthesis, and is classified as essential under standard laboratory conditions (65, 66). Because complete gene knockout is lethal, CRISPRi provides a critical advantage by enabling partial repression of ERG9 expression, allowing us to investigate its contribution to FA resistance in a viable cellular background (65, 66).

In contrast, other genes in the pathway, specifically *ERG25*, were significantly depleted, underscoring that full ERG25 function is essential for survival under FA-induced stress (Fig. 2A).

To assess the role of ERG9 in FA susceptibility, we first generated an ERG9 CRISPRi strain by cloning a single ERG9-targeting gRNA (Table S5) into the ampl43 plasmid (Addgene #161830). As a control, we used an empty vector (E.V.) strain lacking a gRNA to ensure that observed effects were due to gene silencing rather than plasmid expression. Both plasmids were transformed into the BY4741 WT strain. Reverse transcription-quantitative polymerase chain reaction (RT-qPCR) analysis confirmed efficient ERG9 silencing, revealing a 77% reduction in ERG9 mRNA levels upon expression of the ERG9-targeting gRNA (Fig. 2B).

To assess the phenotypic impact of ERG9 silencing, we conducted growth assays in liquid culture, comparing the optical density (O.D.) of the ERG9 CRISPRi strain to the E.V. control under FA treatment. ERG9 silencing conferred a significant increase in FA resistance, as reflected by a higher O.D. compared to the control strain (Fig. 2C). Additionally, spot dilution (drop) assays further confirmed that ERG9 knockdown enhanced FA resistance, with the ERG9 CRISPRi strain displaying robust growth at FA concentrations that inhibited the control strain (Fig. 2D; Fig. S4).

To investigate whether ERG9 silencing affects the expression of upstream genes in the ergosterol biosynthesis pathway, we performed RT-qPCR analysis on nine key upstream genes involved in sterol biosynthesis. While most genes exhibited no significant change in expression following ERG9 silencing (ERG9 CRISPRi), we observed a twofold increase in HMG1 and HMG2 mRNA abundance, which encode rate-limiting enzymes in sterol precursor synthesis (Fig. 2E).

### Proteomic profiling of *Cochliobolus heterostrophus* reveals molecular adaptations to ferulic acid

To complement the CRISPRi findings in yeast, we conducted a comprehensive proteomic analysis of strains of a plant pathogenic fungus, *Cochliobolus heterostrophus,* that experimentally evolved for resistance to FA by *in vitro* evolution. Four independent resistant lineages were generated by gradual adaptation to increasing FA concentrations, exhibiting enhanced tolerance compared to the wild-type parental strain.

**Fig 3:**
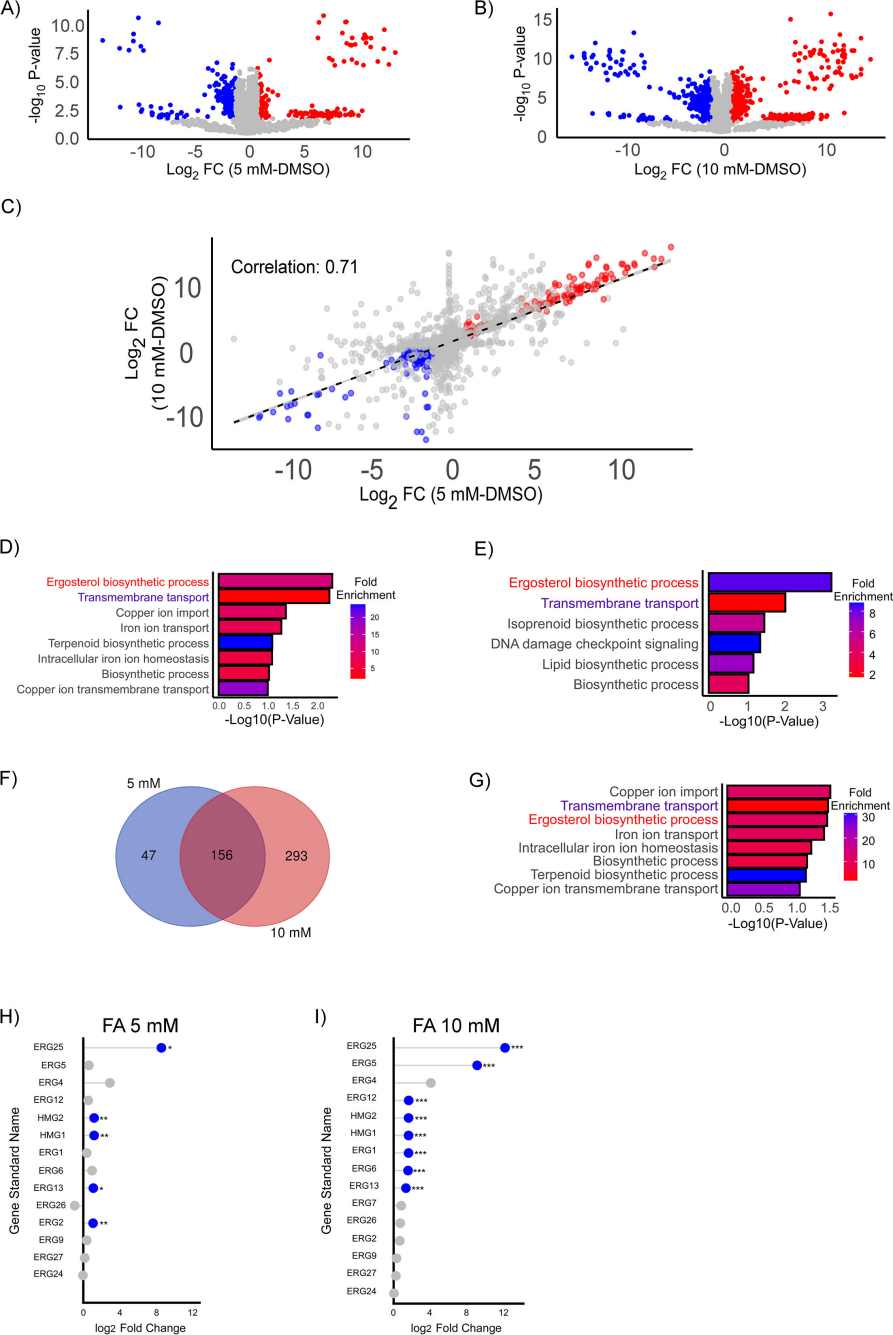
Proteomic analysis of FA-resistant *Cochliobolus heterostrophus* strains. (**A**) Volcano plot showing differential protein abundance between resistant strains treated with 5 mM FA and DMSO control. (**B**) Volcano plot showing differential protein abundance between resistant strains treated with 10 mM FA and DMSO control. Proteins significantly enriched (red) or depleted (blue) were identified using thresholds of −log₁₀(*P* value) >1.3 and |log₂(fold-change)| >1. (**C**) Scatter plot showing correlation of log₂ fold changes between 5 mM FA and 10 mM FA treatments (R = 0.71), indicating a conserved proteomic response. (**D–E**) Functional enrichment analysis of significantly enriched proteins for (**D**) 5 mM FA versus DMSO and (**E**) 10 mM FA versus DMSO, showing −log₁₀(*P* value) and fold enrichment for selected biological processes. (**F**) Venn diagram illustrating the overlap of enriched proteins between 5 and 10 mM FA treatments. (**G**) Functional enrichment analysis of proteins commonly enriched under both 5 and 10 mM FA conditions. **(H–I)** Lollipop plots show log₂ fold changes in protein abundance for ergosterol biosynthesis genes in response to FA treatment, compared with DMSO control. Panel H represents treatment with 5 mM FA; panel I shows 10 mM FA. Blue indicates significant upregulation (log₂ FC > 1, *P* < 0.05); gray indicates non-significant changes. Asterisks denote significance: *P* < 0.05 (*), *P* < 0.01 (**), *P* < 0.001 (***).

To characterize the underlying molecular adaptations, comparative proteomic analyses were performed on resistant strains exposed to high (5 mM) and extreme (10 mM) FA treatment. Protein expression profiles of resistant strains treated with FA were compared to DMSO-treated controls (Fig. 3A and B; Table S4). Both FA conditions revealed extensive sets of differentially enriched and depleted proteins relative to the control.

To assess the consistency of proteomic responses across FA concentrations, we plotted log₂ fold changes for proteins in 5 mM versus 10 mM FA treatments (Fig. 3C). A strong positive correlation (R = 0.71) indicated a conserved proteomic adaptation established during high exposure and maintained under extreme FA stress.

Functional enrichment analysis of GO terms (67) (Fig. 3D and E) revealed that ergosterol biosynthesis represented the most significantly upregulated process in both comparisons.

To enable cross-species functional interpretation of antifungal responses, we first identified *S. cerevisiae* homologs of *Cochliobolus heterostrophus* proteins using BLASTP-based sequence similarity analysis. The *C. heterostrophus* proteome, derived from the FA-resistant strains, was aligned against the reference *S. cerevisiae* S288c proteome (UniProtKB) using a local BLAST+ setup. For each query protein, the top yeast hit (lowest e-value) was retained, yielding a high-confidence ortholog mapping set. The top yeast homologs were annotated with gene names and SGD IDs, allowing integration with ergosterol biosynthesis genes. Lollipop plots show log₂ fold changes in protein abundance following treatment with 5 mM (Fig. 3H) and 10 mM (Fig. 3I) FA, relative to the DMSO control. Five multiple core ergosterol biosynthesis enzymes were significantly upregulated under 5 mM FA, including ERG25, HMG1/2, ERG13, and ERG2. Under 10 mM FA, the effect was more pronounced, with eight proteins significantly enriched, including ERG25, ERG5, ERG12, HMG1/2, ERG1, ERG6, and ERG13 (Fig. 3H and I). These results highlight a coordinated upregulation of multiple enzymes across the mevalonate and late sterol branches of the pathway in response to FA. Notably, ERG25 (N4WWQ4) exhibited particularly high induction, with log₂ fold changes of 8.50 at 5 mM FA and 12.13 at 10 mM FA. These findings align with results from our genome-wide CRISPRi screen in *S. cerevisiae* (Fig. 2A).

In addition to ergosterol biosynthesis, transmembrane transport processes were prominently enriched, underscoring the critical role of transporter proteins in mitigating FA toxicity. ABC transporters were significantly upregulated, with CDR4 (UniProt ID: N4X608) displaying the highest induction (log₂ fold-change = 8.03 at 5 mM FA; 8.05 at 10 mM FA). Similarly, a major facilitator superfamily (MFS) domain-containing protein, N4WZP9, homologous to *Candida albicans* FLU1, exhibited strong upregulation (log₂ fold-change = 5.96 at 5 mM FA; 4.96 at 10 mM FA), highlighting the role of efflux mechanisms in FA resistance (Fig. 3D and E).

Analysis of protein overlap between 5 and 10 mM FA treatments (Fig. 3F) identified 156 shared enriched proteins, indicating the existence of a conserved core proteomic program established early during FA adaptation and maintained under increased FA concentrations. In contrast, proteins uniquely enriched at 10 mM FA may represent additional stress adaptations required to cope with elevated FA exposure.

Functional enrichment analysis of proteins commonly enriched at both 5 and 10 mM FA (Fig. 3G) further emphasized the upregulation of membrane-associated biological processes, including sterol biosynthesis, transmembrane transport, and ion homeostasis. These results suggest that FA resistance involves the coordinated activation of membrane remodeling and efflux transport.

### Evaluation of synergistic antifungal effects of ferulic acid and fluconazole against *Candida albicans* and *Candida parapsilosis*

**Fig 4:**
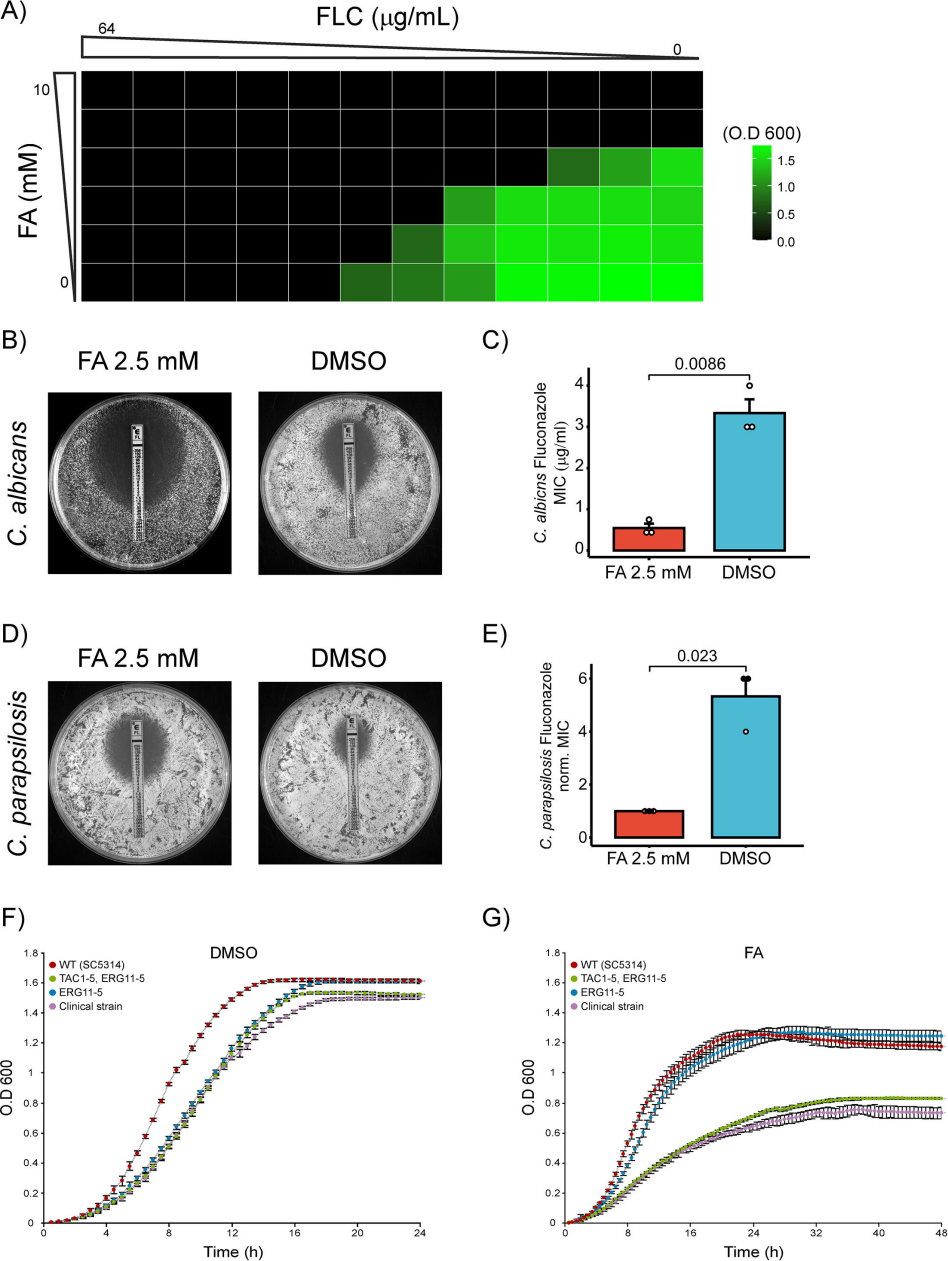
Synergistic antifungal effects of ferulic acid and fluconazole against *C. albicans* and *Candida parapsilosis.* (**A**) Heatmap of a checkerboard assay showing growth inhibition of *C. albicans* SC5314 in response to combinations of FA and FLC. OD₆₀₀ values are color-coded, with darker shading representing stronger inhibition. (**B**) Strip-diffusion assay showing the effect of FLC (E-test strip) on *C. albicans* in the presence of 2.5 mM FA compared to DMSO (control). Commercial E-test strips containing FLC were applied, and plates were incubated at 37°C for 48 h. (**C**) Bar plot depicting the mean MIC of FLC against *C. albicans* treated with 2.5 mM FA compared to the DMSO control. Error bars represent the SEM from three independent biological repeats, and the *P* value was calculated using a one-tailed *t*-test. (**D**) Strip-diffusion assay showing the effect of FLC on *C. parapsilosis* in the presence of 2.5 mM FA compared to DMSO. The experimental conditions were similar to those in (**A**). (**E**) Bar plot showing the mean MIC of FLC against *C. parapsilosis* treated with 2.5 mM FA compared to DMSO. (**F**) Growth curves for *C. albicans* wild-type (WT, SC5314) and azole-resistant strains in the presence of DMSO. Strains include *TAC1* and *ERG11* hyperactive (green), *ERG11* hyperactive (blue), and a clinical azole-resistant strain (purple). Growth was monitored over 24 h, with optical density (O.D. 600) recorded at regular intervals. (**G**) Growth curves for *C. albicans* strains treated with 1.5 mM FA. Growth was measured over 48 h, with error bars representing SEM from four independent biological repeats.

Based on the CRISPRi screen results, repression of *ERG9* modulates FA susceptibility, suggesting that alterations in sterol metabolism influence the cellular response to FA. This suggests that FA has the potential to act synergistically with other antifungal agents that also disrupt this critical pathway, enhancing their combined antifungal efficacy. To investigate whether FA enhances the antifungal efficacy of FLC, we performed a checkerboard synergy assay using *Candida albicans* SC5314, the wild-type reference strain. In the checkerboard assay, FA and FLC were serially diluted in a 96-well plate, and growth was measured after 48 h using OD₆₀₀. Growth inhibition was assessed relative to the MIC_90_ threshold, defined as 90% of the maximal OD. When tested alone, FA exhibited an MIC_90_ of 5 mM and FLC an MIC_90_ of 4 µg/mL. Under combination treatment, however, FA’s MIC_90_ decreased to 0.625 mM and FLC’s to 0.25 µg/mL. This co-inhibitory concentration yielded a fractional inhibitory concentration (FIC_90_) of 0.125 for FA and 0.0625 for FLC, resulting in a combined FICI_90_ of 0.1875. Since this value is well below the established synergy threshold of 0.5, it indicates a strong synergistic interaction between FA and FLC against *C. albicans* SC5314 (Fig. 4A).

To validate this interaction across species, we performed strip-diffusion MIC assays with *C. albicans* and *C. parapsilosis* in the presence of 2.5 mM FA. The MIC of FLC alone for *C. albicans* ranged between 3 and 6 µg/mL; however, when combined with FA, FLC MIC dramatically decreased to 0.25–0.75 µg/mL, representing an eightfold increase in sensitivity (Fig. 4B and C). Similarly, *C. parapsilosis* displayed a reduction in FLC MIC from 24 to 6 μg/mL in the presence of FA (Fig. 4D and E). These results suggest a synergistic effect between FA and FLC, likely related to FA’s ability to disrupt ergosterol biosynthesis.

Furthermore, growth analyses were performed to assess the response of *C. albicans* strains with known azole resistance mechanisms to FA. Three azole-resistant *C. albicans* strains were tested: (i) a strain harboring engineered hyperactive alleles of TAC1 (TAC1-5, encoding the A736V gain-of-function mutation driving CDR1/CDR2 overexpression) and ERG11 (ERG11-5, encoding the S405F substitution that reduces fluconazole binding); (ii) a strain expressing only ERG11-5 (68); and (iii) a fluconazole-resistant clinical isolate, T101 (69), which was isolated from a patient and exhibits resistance through an undefined mechanism (69).

Interestingly, strains overexpressing both TAC1-5 and ERG11-5, as well as the FLC-resistant clinical isolate, exhibited significantly greater growth inhibition in response to FA compared to the WT strain (SC5314) or the strain overexpressing only ERG11 (Fig. 4F and G). These results highlight the potential of FA to synergize with FLC in targeting azole-resistant *C. albicans* strains, suggesting a promising avenue for enhancing antifungal treatment efficacy.

### *In planta* fungicidal efficacy of ferulic acid against the maize pathogen *Cochliobolus heterostrophus*

**Fig 5:**
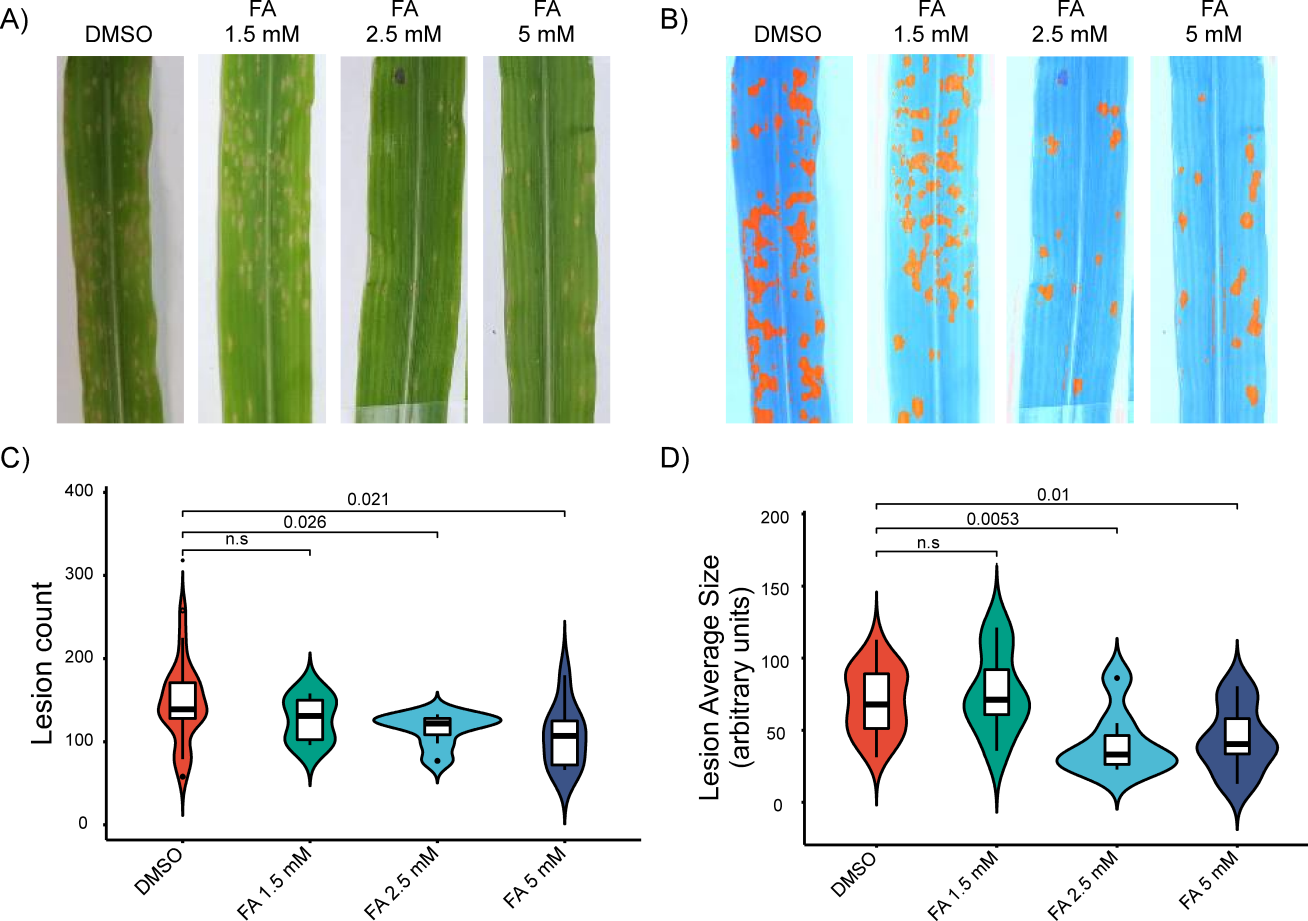
*In planta* antifungal efficacy of FA against *Cochliobolus heterostrophus* on maize leaves. (**A**) Representative images of maize leaves treated with DMSO (control) and FA at concentrations of 1.5, 2.5, and 5 mM. Leaves were inoculated with *C. heterostrophus* spores, and lesion formation was assessed after 72 h. (**B**) ImageJ analysis of lesion development, with lesions detected using the LabKit AI tool. Images show processed data highlighting the detected lesions (orange) for each treatment group. (**C**) Quantification of lesion count per leaf for each treatment group. FA treatment at 2.5 and 5 mM resulted in a significant reduction in lesion count compared to the DMSO control (*P* < 0.05, Wilcoxon rank-sum test). Data presented as median ± interquartile range (IQR). (**D**) Quantification of lesion size in arbitrary units of leaf for each treatment group. FA at 5 mM significantly reduced lesion size compared to the DMSO control (*P* < 0.01, Wilcoxon rank-sum test). Data are presented as median ± IQR.

To investigate the *in planta* antifungal potential of FA, we employed maize (*Zea mays*) as a host system and examined the efficacy of FA in mitigating *Cochliobolus heterostrophus* infection. Maize plants were pre-treated with FA at concentrations of 1.5, 2.5, and 5 mM before being inoculated with *C. heterostrophus* spores. FA is abundant in maize leaves, with an average concentration of about 0.6 mM (70) and an order of magnitude more found in purified cell wall fractions (71). As most of the FA is bound, this is only an estimate of the concentration to which an invading pathogen is exposed, depending on the activity of fungal esterases releasing FA from the host cell wall. The applied concentrations tested are thus chosen to be in the range just above the endogenous levels and are also in the range detected by the pathogen, inducing dephosphorylation of the Hog1/P38 MAP kinase (72). Lesion development on the leaves was monitored 72 h post-inoculation, and lesion parameters (count and size) were quantified using the AI-enhanced ImageJ software, equipped with the LabKit module for high-precision lesion detection and measurement (Fig. 5A and B).

FA treatment significantly reduced both lesion count and lesion size in a dose-dependent manner. At the highest concentration (5 mM), FA-treated leaves displayed a 35% reduction in the median number of lesions compared to the control group treated with DMSO (*P* = 0.021, Wilcoxon rank-sum test) (Fig. 5C), indicating a strong inhibitory effect. In parallel, the size of individual lesions in the 5 mM FA treatment group decreased by 30% relative to the DMSO-treated controls (*P* = 0.01) (Fig. 5D, suggesting that FA inhibits subsequent pathogen proliferation and expansion within the host plant. The 2.5 mM FA treatment similarly showed significant reductions in lesion count (*P* = 0.026) and size (*P* = 0.0053), although the magnitude of these effects was less pronounced than at 5 mM. In contrast, the 1.5 mM FA treatment did not produce statistically significant effects on either lesion count or size, highlighting a potential threshold concentration for FA’s antifungal activity in this system.

## DISCUSSION

### Mechanistic insights from CRISPRi screening

Here, we take advantage of a CRISPRi screen to reveal the molecular mechanisms triggered by FA treatment. The CRISPRi screen results provide a comprehensive view of the cellular response to FA treatment, highlighting both the genes whose downregulation promotes FA resistance and those essential for managing FA-induced stress.

The results suggest that FA treatment triggers a multifaceted stress response, involving significant metabolic reprogramming, activation of autophagy, disruption of protein processing and mRNA stability, and impacts on ribosomal function and cytoskeletal organization. The enrichment and depletion patterns observed provide valuable insights into the complex adaptive strategies employed by cells to mitigate FA-induced damage. These findings emphasize the importance of multiple cellular pathways in managing FA stress, and future research should focus on elucidating the detailed molecular mechanisms underlying these responses and validating the functional roles of the identified genes in FA tolerance.

### Ergosterol biosynthesis pathway and FA resistance

The CRISPRi screen results reveal novel regulatory mechanisms within the well-characterized ergosterol biosynthesis pathway, specifically implicating ERG9 silencing in FA resistance (Fig. 2A). The CRISPRi screen identified ERG9 as the most significant determinant of FA resistance (Fig. 1C and E), highlighting ERG9 silencing as a novel regulatory mechanism within the ergosterol biosynthesis pathway, which is a major target of antifungal drugs. ERG9 functions as a farnesyl-diphosphate farnesyl transferase, crucial for catalyzing the synthesis of squalene by linking two farnesyl pyrophosphate molecules within the sterol biosynthesis pathway (65, 66). In contrast, ERG25 repression led to growth inhibition under FA treatment. These opposing phenotypes highlight distinct mechanistic contributions of early versus late sterol biosynthesis genes to FA sensitivity.

Moreover, ERG9 silencing resulted in a twofold increase in HMG1 and HMG2 mRNA abundance. These paralogs encode HMG-CoA reductase, catalyzing the rate-limiting step of sterol biosynthesis (73, 74) (Fig. 2E). This highlights a previously uncharacterized regulatory link within the established ergosterol biosynthesis pathway. This upregulation could be a compensatory response to maintain sufficient flux through the sterol biosynthesis pathway despite the downregulation of *ERG9*. The observed upregulation of HMG1/2 suggests a compensatory response in sterol metabolism following ERG9 repression. These findings imply that FA susceptibility may be influenced by disruptions to the ergosterol biosynthesis pathway, though a molecular target remains to be determined. However, our data indicate that FA may act on previously uncharacterized enzymes within this pathway, distinguishing it from conventional antifungal agents.

ERG9 functions as a key branch-point enzyme in the mevalonate pathway, where acetyl-CoA is converted into mevalonate and subsequently into farnesyl pyrophosphate (FPP). FPP acts as a precursor for various critical cellular processes, including the biosynthesis of dolichols, ubiquinone, heme A, isoprenylated proteins, and ergosterol (75). We hypothesize that ERG9 silencing may redirect metabolic flux toward alternative isoprenoid pathways, such as ubiquinone biosynthesis; however, this proposed shift requires direct validation through sterol and isoprenoid metabolite profiling. Additionally, the CRISPRi screen results identify Puf3 as a key factor in the cellular response to FA treatment (Fig. S3). Puf3 binds to and promotes the degradation of mRNAs encoding specific nuclear-encoded mitochondrial proteins. Additionally, Puf3 acts as a translational repressor for genes linked to oxidative stress and plays a key regulatory role in ubiquinone (CoQ) biosynthesis (76–80). These observations suggest that the FA resistance conferred by ERG9 silencing involves perturbations in both ergosterol biosynthesis and mitochondrial function.

The ergosterol biosynthesis pathway is the primary target of widely used antifungal drugs, including azoles, yet our findings identify novel regulatory elements within this pathway that modulate FA resistance. A second small-molecule inhibitor may potentiate azole activity against resistant strains (81). This was recently shown for *C. albicans,* where a screen for such potentiators led to 1,4-benzodiazepines (37). Our observations raise the possibility that HMG-CoA reductase might be a target of FA, potentially opening a new avenue to enhance azole efficacy using relatively non-toxic plant-derived antifungals. Mutations in the sterol-sensing domain of *A. fumigatus* HMG-CoA confer resistance to triazoles (82). Although the binding site of FA remains unknown, we speculate that it might interact with the sterol-sensing domain of HMG-CoA reductase.

The proteomic profiling of FA-resistant *Cochliobolus heterostrophus* strains reinforces and extends the findings from the CRISPRi screen in *S. cerevisiae*. Comparative analysis revealed a consistent upregulation of ergosterol biosynthesis enzymes, including ERG2, ERG11, ERG12, ERG13, ERG1, and HMG1/2, with particularly strong induction of ERG25. These observations mirror the genetic sensitivity identified in yeast and underscore the centrality of sterol metabolism in mediating FA resistance.

Importantly, the proteomic data revealed that FA resistance is not limited to sterol pathway remodeling. Enrichment of transmembrane transport processes, especially ABC transporters, along with pathways involved in ion homeostasis, suggests that FA resistance involves a coordinated adaptation that integrates membrane protection and efflux mechanisms.

The convergence of CRISPRi and proteomic results across two phylogenetically distinct fungal species, *S. cerevisiae* and *C. heterostrophus*, supports the notion that FA resistance mechanisms are conserved across fungal pathogens. These findings advance our understanding of how fungi respond to plant-derived antifungal compounds and position ergosterol biosynthesis as a central adaptive hub, not only in model systems but also in agriculturally relevant fungal pathogens.

### Ferulic acid synergy with fluconazole

The mechanism of FA action predicted from our results suggests its potential use as a clinical antifungal agent against human pathogenic fungi, with previous studies confirming its minimal cytotoxicity compared to the therapeutic concentration needed to combat *Candida* spp. The MIC of FA against *C. albicans* is 40 µg/mL (59). Importantly, at a concentration of 300 µg/mL, FA has no adverse effects on the cell count or viability. Furthermore, FA exhibits negligible toxicity to mammalian cell lines at 500 µg/mL (83). We demonstrate that FA enhances FLC efficacy, suggesting a promising strategy for overcoming antifungal resistance (Fig. 4A through E). Checkerboard synergy testing (Fig. 4A) revealed that the MIC₉₀ of FA alone was 5 mM and for FLC was 4 µg/mL, while in combination these values decreased to 0.625 mM and 0.25 µg/mL, respectively. The corresponding fractional inhibitory concentration index values were FIC₉₀ (FA) = 0.125 and FIC₉₀ (FLC) = 0.0625, yielding a FICI₉₀ of 0.1875, indicative of strong synergy.

Additionally, the TAC1-5/ERG11-5 overexpressing strain and the clinical FLC-resistant isolate T101 exhibited significantly greater growth inhibition upon FA exposure compared to the wild-type strain (SC5314), while the strain overexpressing only ERG11-5 showed no comparable sensitivity (Fig. 4F and G). This suggests that overexpression of ABC transporters may contribute to FA hypersensitivity in azole-resistant strains.

Broberg et al. reported that FA can induce the expression of ABC and MFS transporter genes in *C. heterostrophus* (84), raising the possibility that FA modulates transporter regulation in these strains. Future work should assess whether FA alters CDR1/CDR2 expression or efflux activity in resistant and wild-type *Candida* under FA exposure. These findings highlight FA’s potential against azole-resistant strains, though further mechanistic studies are needed to confirm its interaction with drug efflux pathways.

### In planta antifungal efficacy of ferulic acid

Following the demonstration of FA’s antifungal effects *in vitro*, we sought to evaluate its *in planta* efficacy as an agricultural antifungal agent. FA’s *in vitro* antifungal activity has been previously reported against pathogens such as *Fusarium verticillioides, Fusarium graminearum,* and *Sclerotium rolfsii* (85–88). Additionally, Martínez-Fraca et al. found that *Fusarium verticillioides* colonization was significantly lower in maize genotypes with higher ferulic acid levels in the maize seeds’ pericarp tissue compared to those with lower levels (56). However, to the best of our knowledge, no *in planta* experiments evaluating FA’s antifungal efficacy had been published prior to this study.

Our results highlight the *in planta* antifungal efficacy of FA against the maize pathogen *Cochliobolus heterostrophus*, demonstrating a dose-dependent reduction in lesion count and size (Fig. 5). Interestingly, FA affects both lesion count and size (Fig. 5C and D). This suggests that FA likely impairs fungal growth and expansion within the plant. These findings indicate that FA’s antifungal properties, established *in vitro*, extend to *in planta* applications as well, reinforcing its role as a potent antifungal agent in plant systems. The abundance of FA might be increased by breeding crop plants for this trait or by applying FA.

Moreover, the synergistic effects of FA with azole antifungals such as FLC, as observed in clinical fungi, suggest that similar synergies could be explored in agricultural contexts, possibly with agricultural azoles such as tebuconazole or prothioconazole. Future research should prioritize field trials and molecular studies to further elucidate the specific mechanisms by which FA impacts fungal pathogens and to evaluate its broader application in crop protection strategies.

### Conclusions

This study employed both a genome-wide CRISPRi screen in yeast and comprehensive proteomic profiling of FA-resistant *C. heterostrophus* to delineate the molecular mechanisms underlying the antifungal activity of FA. The screen identified multiple cellular pathways affected by FA treatment, including ergosterol biosynthesis, mitochondrial function, and mRNA stability. Notably, *ERG9*, encoding squalene synthase, was identified as a pivotal determinant of FA resistance, where its downregulation significantly increased FA tolerance. Likewise, proteomic analysis of resistant *C. heterostrophus* strains confirmed the upregulation of multiple ergosterol biosynthesis enzymes. Additionally, the RNA-binding protein Puf3 emerged as a key regulator of the FA stress response, potentially influencing mitochondrial protein turnover and modulating oxidative stress responses. FA exposure was also associated with disruptions in autophagy, vacuolar ATPase activity, and ribosomal biogenesis. Importantly, FA exhibited synergistic antifungal activity with FLC, markedly reducing the MIC required to inhibit *Candida* spp. Moreover, *C. albicans* strains with azole resistance show increased sensitivity to FA treatment.

Additionally, in our plant model, FA demonstrated *in planta* antifungal efficacy against *Cochliobolus heterostrophus*, significantly reducing lesion count and size in a dose-dependent manner. This suggests that FA could inhibit both the establishment and expansion of fungal infections in plants, reinforcing its potential as an eco-friendly antifungal agent in agricultural settings. These results elucidate key aspects of FA’s antifungal mode of action and highlight its potential as both a therapeutic agent in combating drug-resistant human pathogens and a sustainable alternative in crop protection.

## MATERIALS AND METHODS

### Strains, growth conditions, and plasmids

*S. cerevisiae* inducible CRISPRi Library (Addgene #161829) was transformed into DH5α *Escherichia coli,* generating more than 4 M colonies. The parental *S. cerevisiae* strain for all studies is BY4741 (Mat a, his3∆1, leu2∆0, met15∆0, ura3∆0); an S288C-derivative laboratory strain. Cultures were usually grown in liquid or on plates of SCD (1× Synthetic Complete Dropout medium with 2% glucose).

For growth curve analysis, overnight stock cultures (5 mL) were grown at 30°C with orbital shaking at 125 rpm. Before initiating the growth experiments, 150 µL of each overnight culture was diluted to 7.5 mL with fresh 1× SCD medium. Optical density (OD600) measurements were taken every 30 min in continuous orbital-shaking mode, with a shaking speed set to slow and a frequency of 559 (1 mm amplitude). Temperature control was maintained at 30°C, unless stated otherwise. Cell growth was assessed via light scatter measurements at 600 nm, and absorbance readings were recorded kinetically using an Agilent BioTek Synergy H1 multimode reader. The reader was controlled, and data were collected using an Agilent BioTek Gen5 microplate reader and imager software.

### *S. cerevisiae* transformation

BY4741 cells in early exponential phase were collected (50 mL) by centrifugation at 4,000 RPM for 4 min at 25°C, washed once with sterile water, and pelleted at 4,000 RPM for 4 min at 25°C. Pellets were resuspended in 0.4 mL of 0.1 M LiAc, and 100 µL per transformation reaction was used. Each 100 µL fraction was pelleted and suspended in 36 µL 1 M LiAc, 5 µL ssDNA (10 mg/mL), and 64 µL plasmid or 100 µL donor DNA from PCR amplification and 240 µL 50% PEG. Samples were incubated for 30 min at 30°C, followed by 20 min at 42°C. Dilutions were plated on SCD-His agar plates to estimate the transformation efficiency, indicating a yield of 482–332 K independent transformants. The rest of the transformation was used to inoculate 100 mL of SCD-His media and grown for ~24 h at 30°C with shaking, at which point the OD600 increased roughly fourfold. A new 100 mL SCD-His culture was inoculated with 400 µL of this culture, and growth at 30°C with shaking was continued overnight. Three aliquots of 25 mL each were taken for yeast plasmid DNA extractions.

### Next-generation sequencing and data analysis

Plasmids were extracted using Zymoprep Yeast Plasmid Miniprep I from three independent biological replicates. The extracted plasmids were amplified using AMP-seq primers in the Supplementary Data S5 using Platinum SuperFi II Green PCR Master Mix following the manufacturer’s protocol. Each 25 µL PCR reaction consisted of 100 ng of plasmid library. The PCR conditions were: 98°C for 2 min for initial denaturing, then 98°C for 30 s, 54°C for 30 s, and 72°C for 30 s for five cycles, then 98°C for 30 s, 64°C for 30 s, and 72°C for 30 s for 15 cycles, then 72°C for 2 min. Amplicon libraries were constructed simultaneously according to Illumina 16S Metagenomic Sequencing Library Preparation, starting from PCR clean-up. An equal volume of the amplicon PCR reaction was taken from all samples for the PCR clean-up. Library QC was performed by measuring library concentration using Qubit with Equalbit dsDNA HS Assay Kit (Vazyme, cat no. EQ121) and size determination using the TapeStation 4200 with the D1000 kit (cat no. 5067-5582). All libraries were mixed into a single tube with equal molarity. The sequencing data were generated on Illumina NextSeq2000, using P2 100 cycles (Read1-100; Index1-8; Index2-8; Read2-0) (Illumina, cat no. 20046811). Cutadapt was employed for sequence trimming, followed by gRNA counting and statistical significance of each gRNA using MAGeCK count. MAGeCKs test was used according to the MAGeCK pipeline (89, 90). Plots and visualizations were generated in R (v4.3.2) using the ggplot2 package.

### Reverse transcription-quantitative polymerase chain reaction

For RT-qPCR analysis, RNA was reverse-transcribed using the qScript cDNA Synthesis Kit following the manufacturer’s protocol. Gene-specific expression levels were quantified in triplicate using a 15 µL reaction volume with PerfeCTa SYBR Green FastMix ROX in a two-step RT-PCR method, as per the manufacturer’s instructions. Primers for the target genes were utilized (Table S5). Data analysis was conducted using the QuantStudio 1 Real-Time PCR System. Fold change was determined using either the 2^−(ΔCt)^ or 2^−(ΔΔCt)^ method. All mRNA levels were analyzed from three independent biological replicates, each with three technical replicates. Plots and visualizations were generated in R (v4.3.2) using the ggplot2 package.

### Sample preparation and mass spectrometry

*C. heterostrophus* FA-resistant strains were collected from CMX agar using SDS buffer (5% SDS, 10 mM DTT, 100 mM Tris-HCl pH 7.8), boiled (95°C, 10 min), sonicated (probe, 30 s, level 5), and centrifuged (10,000 × *g*, RT, 10 min). Proteins were precipitated with 80% ice-cold acetone (−20°C, overnight), washed 3× with cold 80% acetone, and resuspended in 8.5 M urea, 400 mM ammonium bicarbonate, 10 mM DTT. Concentrations were estimated by Bradford assay. Samples were reduced (60°C, 30 min), alkylated with 35.2 mM iodoacetamide in 100 mM ammonium bicarbonate (RT, 30 min, dark), and digested with modified trypsin (Promega) in 1.5 M urea, 66 mM ammonium bicarbonate, overnight at 37°C (1:50 enzyme:substrate), followed by a second digestion (4 h, 1:100). Peptides were desalted (Oasis HLB µElution Plate, Waters), dried, and reconstituted in 0.1% formic acid, 2% acetonitrile. Peptides were analyzed by liquid chromatography-tandem mass spectrometry (Exploris 480, Thermo) with Vanquish high-performance liquid chromatography and a 30 cm × 75 µm Reprosil C18-Aqua column. A 120 min gradient (6%–34%) of solvent B (80% acetonitrile + 0.1% formic acid) was followed by ramping to 99% (0.1 min) and hold (11 min) at 0.15 µL/min. MS used full scans (m/z 380–985, 120,000 resolution) and DIA (10 Da windows, 1 m/z overlap, 30,000 resolution). Data were analyzed with DIA-NN v1.9.2 (91, 92) against the *C. heterostrophus*-C4-Uniprot-08-20 database (min peptide length = 7, max 1 missed cleavage, fixed carbamidomethylation of Cys, variable N-terminal acetylation). False discovery rate (FDRs) for peptides/proteins were set at 1%. Statistical analysis used Perseus v2.1.3.0 (93).

### BLAST-based functional homology mapping

To infer functional homologs, *Cochliobolus heterostrophus* race T (strain C4) protein sequences were aligned to the *S. cerevisiae* S288c proteome using BLASTP (BLAST+ v2.14.0). The top yeast hit per fungal protein (e-value < 1e–5) was retained. UniProtKB identifiers were mapped to SGD systematic names and GO terms using UniProt’s batch annotation tool. Merged data sets were filtered to Kyoto Encyclopedia of Genes and Genomes pathway members involved in sterol biosynthesis (sce00100, sce00900, M00102). Plots and pathway visualizations were generated in R (v4.3.2) using the tidyverse, ggplot2, and pathview packages.

### CRISPR/Cas9-mediated recombination and random mutagenesis

Homologous recombination via CRISPR/Cas9 and random mutagenesis followed established protocols (94, 95). Cells were harvested during the early exponential phase (50 mL) by centrifugation at 4,000 RPM for 4 min at 25°C, washed once with sterile water, and pelleted again at 4,000 RPM for 4 min at 25°C. The resulting pellets were resuspended in 0.4 mL of 0.1 M LiAc, and 100 µL per transformation reaction was utilized. Each 100 µL fraction was then pelleted and suspended in 40 µL sterile water, 36 µL 1 M LiAc, 25 µL ssDNA (2 mg/mL), 4 mL (2 µg) plasmid, 30 µL donor DNA (100 mM stock) (Table S3), and 240 µL 50% PEG. Samples were subjected to a 30 min incubation at 30°C followed by 15 min at 42°C. Transformation mixtures were plated on YPG supplemented with 100 µg/mL Hygromycin B plates. Positive colonies were harvested after 3 days and replated on a selection medium for verification.

### Antifungal susceptibility assay

The antifungal susceptibility of *C. albicans* strain SC5314 to FLC, both alone and in combination with FA, was evaluated using commercial drug-embedded E-test strips. The assay was conducted following the manufacturer’s protocol. For the strip diffusion assays, *C. albicans* (4  ×  10^3^) suspended in 0.2 mL of sterile water was spread evenly onto yeast synthetic complete (SCD) medium plates. For the combination treatment, 2.5 mM FA was incorporated into the SCD medium. The antifungal-embedded E-test strips were then applied to the dried agar plates. After 48 h of incubation at 37°C, the MICs were determined by measuring the zones of clearance around the E-test strips. Plots and visualizations were generated in R (v4.3.2) using the ggplot2 package.

### Checkerboard assay

FA and fluconazole (FLC; Sigma-Aldrich) were tested using a 96-well checkerboard broth microdilution assay. *Candida albicans* strains were inoculated in SC medium, and optical density at 600 nm was measured after 48 h incubation at 37°C. The fractional inhibitory concentration index (FICI) was calculated as


$$FICI=\left(\frac{MIC{\;}_{FA}\;\left(combo\right)}{MIC{\;}_{FA}\;\left(alone\right)}\right)+\left(\frac{MIC{\;}_{FLC}\;\left(combo\right)}{MIC{\;}_{FLC}\;\left(alone\right)}\right).$$

### *In Planta* antifungal efficacy of ferulic acid in maize

Plants of the hybrid maize line Royalty were grown for 3 weeks till the fourth leaf emerged in a controlled greenhouse environment at 25°C. The third fully developed leaf of each maize plant was treated by spraying with FA solutions at concentrations of 1.5, 2.5, and 5 mM dissolved in 0.02% Triton X-100 as a surfactant. Control plants were treated with DMSO in 0.02% Triton X-100. After FA treatment, leaves were allowed to dry for 1 h, then inoculated with a *Cochliobolus heterostrophus* (race T strain C4) spore suspension (10⁵ spores/mL) evenly applied by spraying across the leaf surface. Following inoculation, plants were incubated under high humidity for 24 h, followed by ambient greenhouse conditions for 48 h. Lesion development was documented 72 h post-inoculation. Lesion count and size were analyzed using ImageJ software with the LabKit AI tool trained on infected leaf samples to create a classifier for lesion detection. Statistical analysis was performed using the Wilcoxon rank-sum test. Plots and visualizations were generated in R (v4.3.2) using the ggplot2 package.

### Confocal fluorescence microscopy

Cells were induced as described and then fixed with 4% paraformaldehyde in PBST. Following fixation, cells were rinsed three times with PBST. Imaging was performed using a Spinning Disk confocal microscope (Yokogawa CSU-W1, Nikon) equipped with a CFI Plan Apochromat 100× oil immersion objective (N.A. 1.45). GFP signals were captured using 488 nm laser excitation, and emissions were detected with a high-sensitivity sCMOS camera (Photometrics, PRIME-BSI, 95% QE) via NIS software. Images were acquired in Z-stack mode with 0.2–0.4 µm intervals across 8–15 µm. Deconvolution was applied to images of GFP-expressing strains using NIS-elements, and the NIS Denoise AI algorithm was used for noise reduction. All data were collected under consistent imaging conditions, including laser power, optical path, and camera exposure time.

## Data Availability

All data generated or analyzed during this study are available in the supplementary materials. The CRISPRi Amplicon-seq data for all samples can be accessed at the European Nucleotide Archive (ENA) under the primary accession PRJEB95957, with the sample group ERP178710.

## SUPPLEMENTAL MATERIAL

Supplemental Figures mbio.01909-25-s0001.docx Figures S1 to S4.

Captions mbio.01909-25-s0002.docx Captions for supplemental tables.

Table S1 mbio.01909-25-s0003.xlsx sgRNA counts.

Table S2 mbio.01909-25-s0004.xlsx Gene summary.

Table S3 mbio.01909-25-s0005.xlsx GO enrichment analysis.

Table S4 mbio.01909-25-s0006.xlsx Proteomics.

Table S5 mbio.01909-25-s0007.xlsx Oligonucleotides.

ASM does not own the copyrights to Supplemental Material that may be linked to, or accessed through, an article. The authors have granted ASM a non-exclusive, world-wide license to publish the Supplemental Material files. Please contact the corresponding author directly for reuse.
