# Supplementary material for: Genome-wide CRISPRi screen and proteomic profiling identify key genes related to ferulic acid’s antifungal activity
Source: mBio. 2025 Aug 25;16(10):e01909-25. doi: 10.1128/mbio.01909-25 (PMC12505964; doi:10.1128/mbio.01909-25)
Supplement: Supplemental Figures — Figures S1 to S4. [file mbio.01909-25-s0001.docx]

**
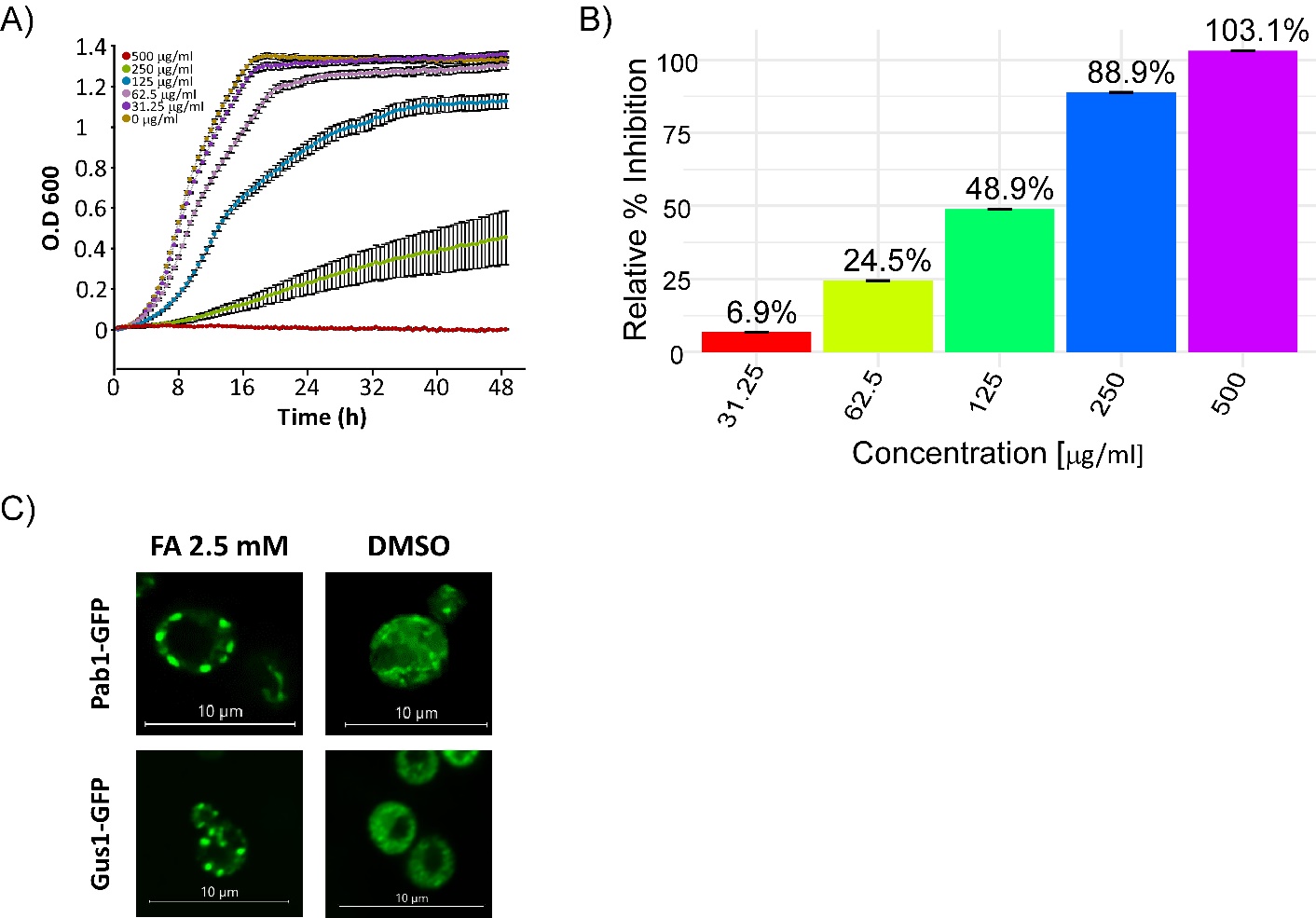
**

**Supplementary Figure 1: Yeast Response to Ferulic Acid (FA) Treatment**

**A**) Growth curves of yeast cells treated with various concentrations of FA (0 to 500 μg/ml). Optical density (O.D 660) was measured over 48 hours. **B**) Relative Inhibition of yeast growth at various concentrations of FA (31.25 to 500 μg/ml). The growth rate at each FA concentration were normalized to DMSO control. **C**) Fluorescence microscopy images showing the localization of Pab1-GFP and Gus1-GFP in yeast cells treated with 2.5 mM FA and DMSO control. Scale bars represent 10 μm.


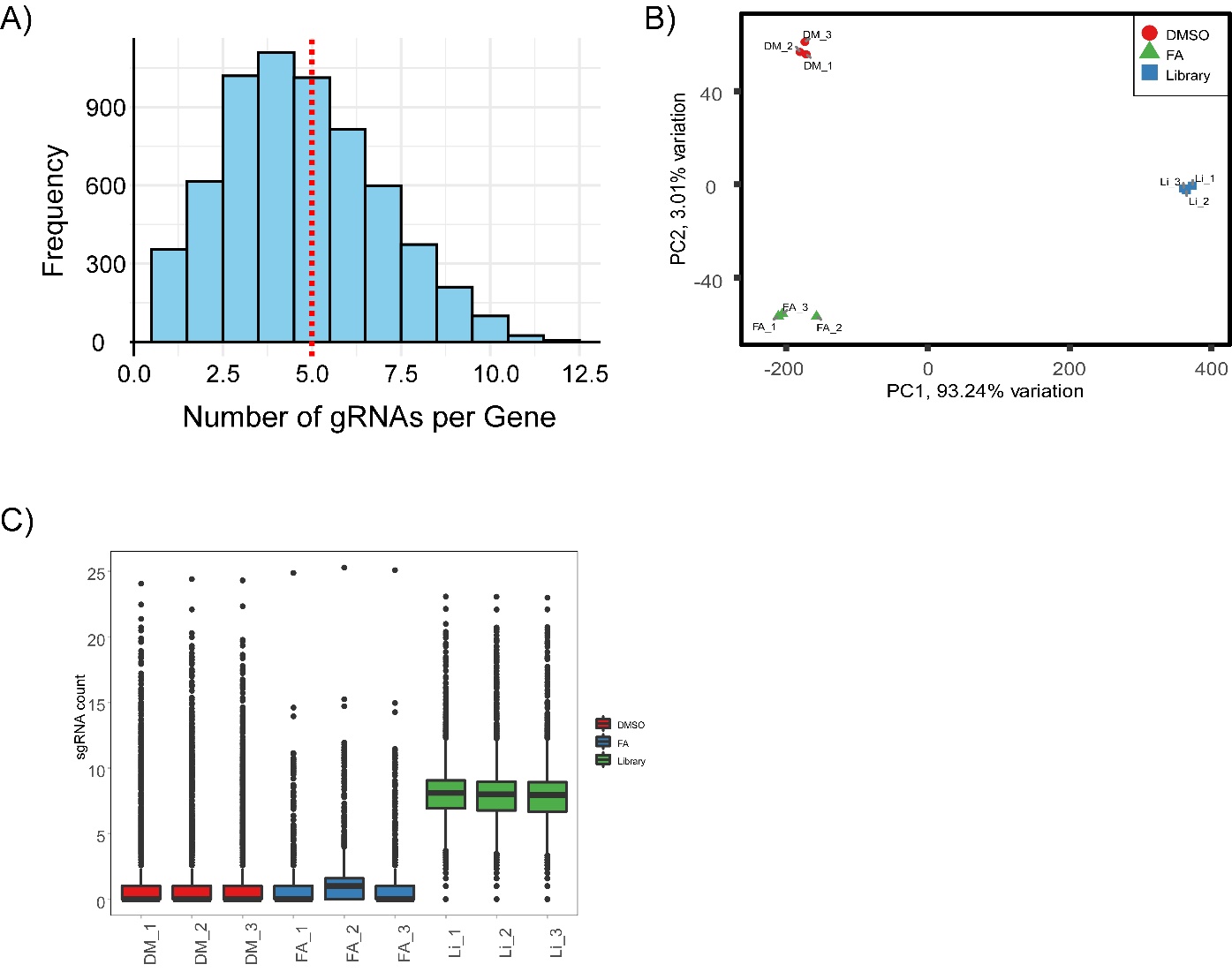


**Supplementary Figure 2: CRISPRi-seq Analysis of S. cerevisiae Treated with Ferulic Acid (FA) and DMSO Control**

**A)** Histogram depicting the number of gRNAs per gene in three library replicates. The dashed red line denotes the median. **B)** Principal Component Analysis (PCA) plot showing the clustering of samples treated with DMSO (red circles), FA (green triangles), and the initial library (blue squares). **C)** Box plots representing the distribution of sgRNA counts across different samples (DMSO, FA and initial library). The FA-treated samples show a reduced median sgRNA count and increased variability compared to the DMSO control.

**
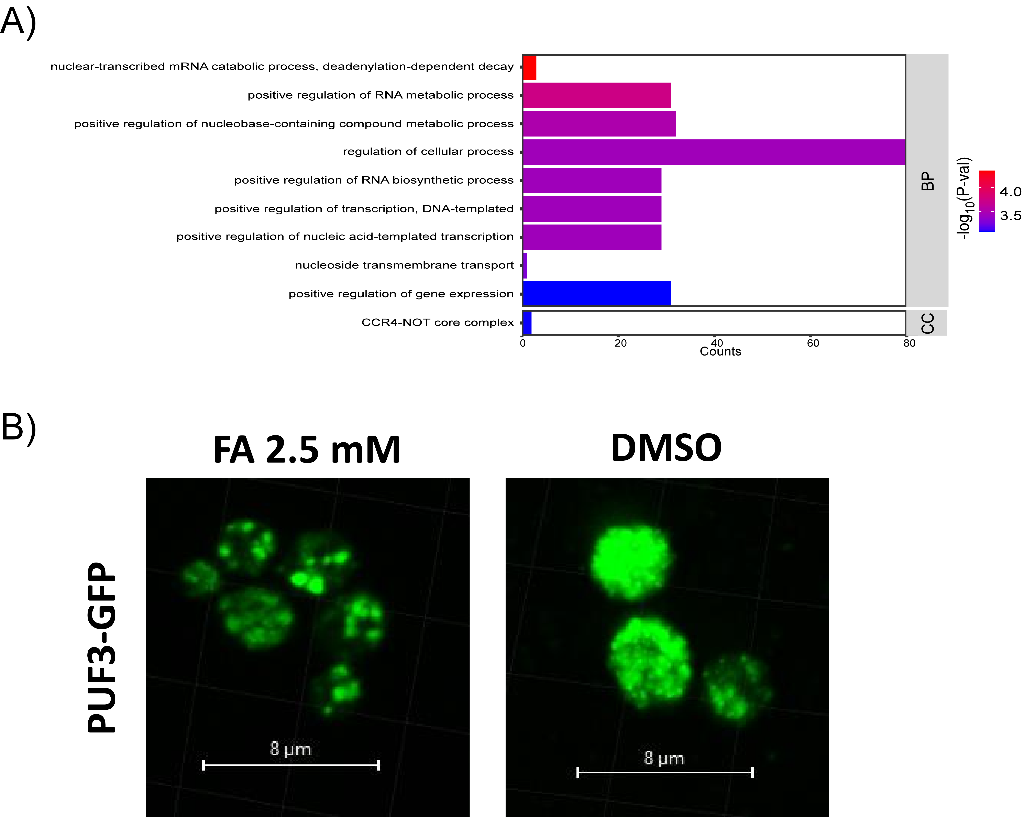
**

**Supplementary Figure 3: Gene Ontology (GO) term enrichment analysis and PUF3-GFP Localization Under FA Treatment**

**A**) Gene Ontology (GO) term enrichment analysis of CRISPRi-seq depleted genes, highlighting biological processes regulation that are crucial for FA resistance. **B**) Fluorescence microscopy images showing the localization of PUF3-GFP in cells treated with 2.5 mM FA (left) and DMSO (right). Scale bars represent 8 μm.


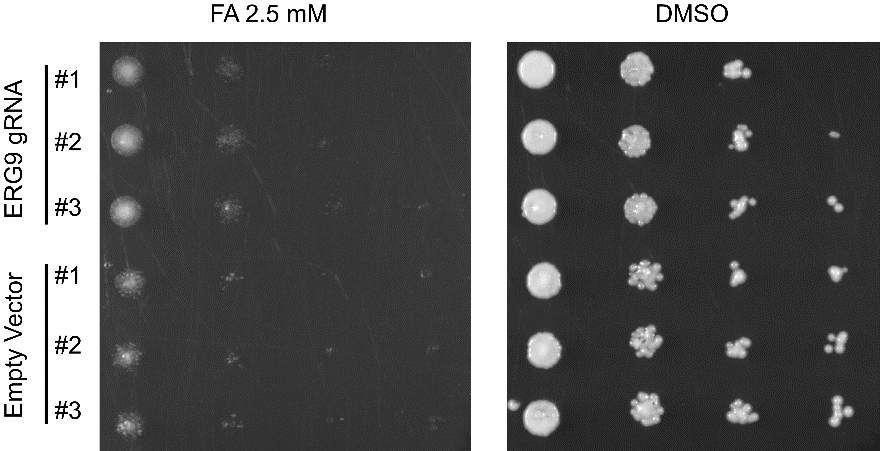


**Supplementary Figure 4: Yeast drop assay**

Yeast drop assay evaluating FA resistance in the ERG9 CRISPRi strain compared to the Empty Vector control. Growth was observed over 48 hours in the presence of DMSO or 2.5 mM FA.
