# Supplementary material for: Genome-wide CRISPRi screen and proteomic profiling identify key genes related to ferulic acid’s antifungal activity
Source: mBio. 2025 Aug 25;16(10):e01909-25. doi: 10.1128/mbio.01909-25 (PMC12505964; doi:10.1128/mbio.01909-25)
Supplement: Captions — for supplemental tables. [file mbio.01909-25-s0002.docx]

**Supplementary Table S1. sgRNA Counts for CRISPRi Screen under Ferulic Acid and DMSO Conditions**
This table provides raw count data for each sgRNA used in the genome-wide CRISPRi screen performed in *Saccharomyces cerevisiae* under Ferulic Acid and DMSO treatment conditions, as well as in the initial input library. These data were used as input for downstream MAGeCK analysis to identify gene-level depletion and enrichment under treatment conditions.

**Supplementary Table S2. Gene Summary from CRISPRi Screen Analysis**
This table provides the gene-level summary statistics from the genome-wide CRISPRi screen conducted in *Saccharomyces cerevisiae* under Ferulic Acid treatment. The analysis was performed using MAGeCK's gene-level negative and positive selection tests based on sgRNA depletion and enrichment patterns.

**Supplementary Table S3. Gene Ontology (GO) Enrichment Analysis of CRISPRi-Depleted Genes Using GOrilla**
This table summarizes the results of a Gene Ontology (GO) enrichment analysis performed using the GOrilla tool on genes significantly depleted in the CRISPRi screen under Ferulic Acid treatment. The analysis identifies GO terms statistically overrepresented in the dataset, providing insight into the biological functions, processes, and cellular components associated with Ferulic Acid sensitivity.

**Supplementary Table S4. Differential Proteomic Analysis of Ferulic Acid–Resistant *Cochliobolus heterostrophus* Strains**
This table presents quantitative proteomic data comparing Ferulic Acid (FA)–resistant *C. heterostrophus* strains under baseline (DMSO) and FA treatment conditions. The analysis highlights proteins whose abundance is significantly altered in response to FA exposure, providing insight into molecular mechanisms associated with resistance and stress adaptation.

**Supplementary Table S5. Oligonucleotides Used for CRISPRi, Amplicon Sequencing, and RT-qPCR**
This table lists all oligonucleotides employed in this study for CRISPR interference (CRISPRi) experiments, Illumina-compatible amplicon sequencing, quantitative gene expression analysis, and gRNA cloning.
